# Missing Data in Clinical Research: A Tutorial on Multiple Imputation

**DOI:** 10.1016/j.cjca.2020.11.010

**Published:** 2021-09

**Authors:** Peter C. Austin, Ian R. White, Douglas S. Lee, Stef van Buuren

**Affiliations:** a Institute for Clinical Evaluative Sciences, Toronto, Ontario, Canada; b Institute of Health Policy, Management and Evaluation, University of Toronto, Ontario, Canada; c Sunnybrook Research Institute, Toronto, Ontario, Canada; d Medical Research Council Clinical Trials Unit, University College London, London, United Kingdom; e Department of Medicine, University of Toronto, Toronto, Ontario, Canada; f Peter Munk Cardiac Centre and University Health Network, Toronto, Ontario, Canada; g University of Utrecht, Utrecht, The Netherlands; h Netherlands Organisation for Applied Scientific Research, Leiden, The Netherlands

## Abstract

Missing data is a common occurrence in clinical research. Missing data occurs when the value of the variables of interest are not measured or recorded for all subjects in the sample. Common approaches to addressing the presence of missing data include complete-case analyses, where subjects with missing data are excluded, and mean-value imputation, where missing values are replaced with the mean value of that variable in those subjects for whom it is not missing. However, in many settings, these approaches can lead to biased estimates of statistics (eg, of regression coefficients) and/or confidence intervals that are artificially narrow. Multiple imputation (MI) is a popular approach for addressing the presence of missing data. With MI, multiple plausible values of a given variable are imputed or filled in for each subject who has missing data for that variable. This results in the creation of multiple completed data sets. Identical statistical analyses are conducted in each of these complete data sets and the results are pooled across complete data sets. We provide an introduction to MI and discuss issues in its implementation, including developing the imputation model, how many imputed data sets to create, and addressing derived variables. We illustrate the application of MI through an analysis of data on patients hospitalised with heart failure. We focus on developing a model to estimate the probability of 1-year mortality in the presence of missing data. Statistical software code for conducting MI in R, SAS, and Stata are provided.

## Résumé

Les données manquantes sont un phénomène courant dans le domaine de la recherche clinique, qui survient lorsque les résultats pour des variables d'intérêt ne sont pas mesurés ou consignés pour tous les sujets d'un échantillon. Les approches courantes adoptées pour pallier les données manquantes comprennent les analyses de cas complètes, dans lesquelles tous les sujets pour lesquels des données sont manquantes sont exclus de l'analyse, et l'imputation par la moyenne, dans laquelle les valeurs manquantes sont remplacées par la valeur moyenne rapportée pour cette variable chez les sujets chez lesquels ces résultats ont été recueillis. Toutefois, dans de nombreux contextes, ces approches peuvent donner lieu à des estimations biaisées des statistiques (p. ex. des coefficients de régression) ou à des intervalles de confiance artificiellement étroits. L'imputation multiple est une approche populaire pour remédier aux données manquantes. Selon cette méthode, des valeurs plausibles multiples pour une variable donnée sont attribuées ou imputées pour chacun des sujets pour lesquels les résultats pour ladite variable sont manquants. Il en résulte la création de multiples groupes de données complètes. Des analyses statistiques identiques sont effectuées à partir de chacun de ces groupes de données complètes, et les résultats sont regroupés pour les différents groupes de données complètes. Cet article offre une introduction à l'imputation multiple, et aborde les difficultés liées à son utilisation, notamment l’élaboration du modèle d'imputation, le nombre de groupes de données imputables à créer, et les variables dérivées qui doivent être considérées. L'application de l'imputation multiple sera illustrée au moyen d'une analyse des données pour des patients hospitalisés atteints d'insuffisance cardiaque. Le modèle suggéré aura pour objectif d'estimer la probabilité de mortalité à 1 an en présence de données manquantes. Les codes pour les logiciels statistiques utilisés pour l'imputation multiple (R, SAS et Stata) sont fournis.

Missing data is a common occurrence in clinical research. Missing data occurs when the values of the variables of interest are not measured or recorded for all subjects in the sample. Data can be missing for several reasons, including: (i) patient refusal to respond to specific questions (eg, patient does not report data on income); (ii) loss of patient to follow-up; (iii) investigator or mechanical error (eg, sphygmomanometer failure); and (iv) physicians not ordering certain investigations for some patients (eg, cholesterol test not ordered for some patients).

Before discussing different ways of addressing the presence of missing data, it is important to understand the conditions under which data are subject to being missing. Rubin developed a framework for addressing missing data and described 3 different missing-data mechanisms.^1^^,^^2^ Data are said to be “missing completely at random” (MCAR) if the probability of a variable being missing for a given subject is independent from both observed and unobserved variables for that subject.^3^ If data are MCAR, then the subsample consisting of subjects with complete (or nonmissing) data is a representative subsample of the overall sample. An example of MCAR is a laboratory value that is missing because the sample was lost or damaged in the laboratory. The occurrence of such events in the laboratory is unlikely to be related to characteristics of the subject. Data are said to be “missing at random” (MAR) if, after accounting for all the observed variables, the probability of a variable being missing is independent from the unobserved data. If physicians were less likely to order laboratory tests for older patients and that was the only factor influencing whether or not a test was ordered and recorded, then missing laboratory data would be MAR (assuming that age was recorded for all patients). Finally, data are said to be “missing not at random” (MNAR) if they are neither MAR nor MCAR. Thus, data are MNAR if the probability of a variable being missing, even after accounting for all the observed variables, is dependent on the value of the missing variable. An example of data that are MNAR is income, in which more affluent subjects, even after accounting for other characteristics, are less likely to report their income in surveys than are less affluent subjects. Unfortunately, one cannot test whether the data are MAR vs MNAR, so one must judge what is plausible using clinical knowledge.^4^^,^^5^

Historically, a popular approach when faced with missing data was to exclude all subjects with missing data on any necessary variables and to conduct subsequent statistical analyses using only those subjects who have complete data (accordingly, this approach is often referred to as “complete case” analysis). When only the outcome variable is incomplete, this approach is valid under MAR and often appropriate.^6^ With incomplete covariates, there are disadvantages to this approach.^2^^,^^4^^,^^7^ First, unless data are MAR, the estimated statistics and regression coefficients may be biased.^4^ Second, even if data are MCAR, with the reduction in sample size there is a corresponding reduction in precision with which statistics and regression coefficients are estimated. Accordingly, estimated confidence intervals will be wider when using complete case analysis than if all the data were used. Moreover, different analyses may use different subsets of the overall sample, so that it is difficult to compare results even within the same paper.

An approach to circumvent the limitations of a complete case analysis is to replace the missing values of variables with plausible values. Such an approach is called “imputation,” because one is imputing a value of the variable for those subjects with missing data on that variable. Historically, a common approach to imputation was “mean-value imputation,” in which subjects for whom a given variable is missing have the missing value replaced with the mean value of that variable among all subjects for whom the variable is present. Thus, subjects who are missing blood pressure have the missing value replaced with the average value of blood pressure among those subjects for whom blood pressure was measured and recorded. A limitation of mean-value imputation is that it artificially reduces the variation in the data set. For example, mean imputation will artificially lower the estimated standard deviation of the variable that includes imputed values.^2^ Furthermore, mean-value imputation ignores multivariate relations between different variables in the sample. For instance, older subjects may have, on average, higher blood pressure than younger subjects. This correlation between age and blood pressure is not taken into account by mean-value imputation.

An alternative to mean value imputation is “conditional-mean imputation,” in which a regression model is used to impute a single value for each missing value.^2^ From the fitted regression model, the mean or expected value, conditional on the observed covariates, is imputed for those subjects with missing data. Thus, assuming that the imputation model regressed blood pressure on age and sex, the same value of blood pressure would be imputed for all subjects of the same age and sex. A modification of conditional-mean imputation draws the imputed value from a conditional distribution whose parameters are determined from the fitted regression model. However, both of these approaches artificially amplify the multivariate relationships in the data. Another limitation is that the imputed values are treated as known with certainty and treated on an equal footing with the values for the same variable for other subjects for whom the variable was observed and recorded and not imputed. Mean-value imputation and conditional-mean imputation are recommended for handling missing values of baseline covariates in randomised trials only.^6^^,^^8^^,^^9^

A popular approach for addressing the issue of missing data is multiple imputation (MI).^1^^,^^10^ MI imputes multiple values for each missing value. This results in the creation of multiple complete data sets in which the missing values have been filled in with plausible values. The analysis of scientific interest is then conducted separately in each of these complete data sets and the results are pooled across the imputed data sets. In this way, MI allows the user to explicitly incorporate the uncertainty about the true value of imputed variables.

The present paper provides an introduction to MI and illustrates its application with the use of a cardiovascular example. The paper is structured as follows. In the next section we introduce MI and discuss several issues related to its implementation. Then (Case Study) we illustrate its application with an example of logistic regression to model mortality in patients with heart failure. Finally (Discussion), we summarise our brief tutorial and direct the interested reader to more detailed and comprehensive discussions of MI.

## Multiple Imputation for Missing Data

In this section we provide an introduction to MI and discuss issues related to its use.

### Multiple imputation using multivariate imputation by chained equations

Fully conditional specification is a strategy for specifying multivariate models through conditional distributions. A specific implementation of this strategy in which every variable is imputed conditional on all other variables is now known as the multivariate imputation by chained equations (MICE)^10^, ^11^, ^12^, ^13^ algorithm. In our description of the algorithm we assume that there are *p* variables, of which *k* are subject to missing data and *p − k* are complete. The algorithm is summarised in Table 1. The process described in steps 3 and 4 is repeated for several cycles to create 1 imputed data set. Standard software uses 5 to 20 cycles by default, and it is rarely necessary to increase these values.^10^^,^^11^ The imputed values obtained after the last cycle are used as the imputed values for the first imputed data set. The entire process is then repeated M times to produce M imputed data sets.

**Table 1:** Multivariate imputation by chained equations (MICE) algorithm for multiple imputation

1. Specify an imputation model for each of the *k* variables that are subject to missing data.
2. For each of the *k* variables that are subject to missing data, fill in the missing values with random draws from those subjects with observed values for the variable in question. Note that these initial imputed values do not respect the multivariate relations in the data and will be overwritten by better imputed values in later stages of the algorithm.
3. For the first variable that is subject to missing data:
a. Regress this first variable on all the other variables using those subjects with complete data on the first variable and observed or currently imputed values of the other variables.
b. The estimated regression coefficients and their variance-covariance matrix (and the estimated variance of the residual distribution if a linear regression model was fit for a continuous variable) are extracted from the regression model estimated in (a).
c. Using the quantities obtained in (b), randomly perturb the estimated regression coefficients in a way that reflects the degree of uncertainty arising from the data.
d. Using the set of perturbed regression coefficients obtained in (c), the conditional distribution of the first variable is determined for each subject with missing data on that variable.
e. A value of the variable is drawn from this conditional distribution for each subject with missing data on the first variable.
4. Repeat step 3 for each of the variables that is subject to missing data. Steps 3 and 4 form 1 cycle of the imputation process for creating 1 imputed data set.
5. Repeat steps 3 and 4 the desired number of times (suggested 5 to 20 cycles). The final imputed values are used as the imputed values in first imputed data set.
6. Repeat steps 2-5 M times to produce M imputed data sets (the choice of M, the number of imputed data sets, is discussed in the section How Many Imputations: How Large Should M Be?).

### Multiple imputation for continuous variables with the use of predictive-mean matching

The imputation process described above uses linear regression and takes the imputed values as random draws from a normal distribution. This has problems if the residuals from the regressions are not normally distributed (eg, if data are skewed) or if relationships are nonlinear (eg, height and age). For example, a variable that can have only positive values (eg, counts) may have imputed values that are negative. One option to address such problems is to transform the variable before imputation so that the transformed variable is approximately normally distributed. For example, the logarithmic transformation, when applied to a positively skewed distribution, can result in a distribution that is more normally distributed. As a last step, one may wish to back-transform imputations into the original scale. A second option to is to draw imputations from the observed values by a technique called predictive-mean matching (PMM).^11^ For a given subject with missing data on the variable in question, PMM identifies those subjects with no missing data on the variable in question whose linear predictors (created using the regression coefficients from the fitted imputation model) are close to the linear predictor of the given subject (created using the regression coefficients sampled from the appropriate posterior distribution, as described above). Of those subjects who are close, one subject is selected at random and the observed value of the given variable for that randomly selected subject is used as the imputed value of the variable for the subject with missing data. Morris et al. suggest that identifying the 10 closest subjects without missing data performs well.^14^ Using the terminology of Morris et al., we refer to the method described in this section as parametric imputation, because the imputed variables are drawn from a parametric distribution.^14^ This is in contrast to PMM, where the imputed variables are drawn from an observed empirical distribution.

### Analyses in the M imputed data sets

Once M complete data sets have been constructed using MI, the statistical analysis of scientific interest is conducted in each of the M complete data sets. That analysis would be the exact analysis that would be conducted in the absence of missing data. Thus, if the analysis model is a logistic regression model in which a binary outcome variable is regressed on a set of predictor variables, that model is fitted in each of the M imputed data sets. The statistics of interest (eg, estimated regression coefficients and their standard errors) are extracted from the analysis conducted in each of the M imputed data sets.

### Rubin’s rules for combining estimates and standard errors across imputed data sets

Once the statistics of interest have been estimated in the M imputed data sets, they are combined using Rubin’s rules.^1^ Let *θ*^(*i*)^ denote the estimated statistic of interest (eg, a regression coefficient) obtained from the analysis in the *i*th imputed data set (*i* = 1,…, M). The pooled estimated of the statistic of interest is $\theta =\frac{1}{M}\sum_{i=1}^{M}\theta^{\left(i\right)}$. The MI estimate of the statistic is simply the average value of the estimated statistic across the M imputed data sets.

Computing the variance of the estimated statistic is more complex, as it requires accounting for the within-imputation uncertainty in the estimated statistic and the between-imputation variation in the estimated statistic. Let *W*^(*i*)^ denote the estimated variance (eg, the square of the estimated standard error) of *θ*^(*i*)^. The average within-imputation variance is defined as $W=\frac{1}{M}\sum_{i=1}^{M}W^{\left(i\right)}$. This is simply the mean estimated variance of the estimated statistic across the M imputed data sets. The between-imputation variance of the estimated statistic is $B=\frac{1}{M-1}{\sum_{i=1}^{M}\left(\theta^{\left(i\right)}-\theta \right)}^{2}$. This quantity reflects the degree to which the estimated statistic varies across the M imputed data sets. The MI estimate of the variance of *θ* obtained with the use of Rubin’s rules is $\mathrm{var}\left(\theta \right)=W+\left[1+\frac{1}{M}\right]B$. This quantity reflects both the average within-imputation variation and the average between-imputation variation in *θ*. Note that when using single imputation, there is no estimate of B, so we are unable to estimate the true variation in the statistic.

### How many imputations: How large should M be?

An important question is how many imputed data sets should be created. Early recommendations were that 3 to 5 imputed data sets were sufficient as long as the amount of missing information was not very high,^1^^,^^3^ while others suggested that often 5 to 10 imputations were required to be sufficient.^7^ These early recommendations were based on the accuracy with which the regression coefficient was estimated compared with its accuracy had it been estimated with an infinite number of imputed data sets. However, analysts are interested not only in estimated regression coefficients (eg, log odds ratios or log hazard ratios), but also in their associated standard errors (which are used in deriving confidence intervals and significance tests). Thus, one wants to estimate not only regression coefficients accurately, but also standard errors.

Ideally, one would select M such that the pooled estimated regression coefficients and standard errors would not vary meaningfully across repeated applications of MI (ie, if the entire process was repeated with M new imputed data sets, one would obtain estimates similar to those obtained using the initial M imputed data sets). The term Monte Carlo error in a given statistic (eg, a regression coefficient or a standard error) refers to the standard deviation of that statistic across repeated applications of MI. When focusing on a single statistic, the Monte Carlo error can be computed as $\sqrt{B/M}$.^11^ White et al. suggested that, as a rule of thumb, the number of imputed data sets should be at least as large as the percentage of subjects with any missing data.^11^ They suggest that this will result in estimates of regression coefficients, test statistics (regression coefficients divided by the standard error), and *P* values with minor variability across repeated MI analyses (ie, the Monte Carlo error will be low). A more advanced method for determining the number of imputations was developed by von Hippel.^15^ Nowadays, computation is cheap and the use of 20 to 100 imputed data sets is common.

### Which variables to include in the imputation model?

Investigators need to distinguish between 2 different statistical models: the imputation model and the analysis model. The imputation model is used for imputing missing data. It is not of direct interest and is only used to provide reasonable imputations. The analysis model holds the quantities that are ultimately of scientific interest and is the focus of the research question. The rules for building imputation and analysis models are very different. It is important to include in the imputation model all the variables that will be included in the analysis model. Failure to include these variables in the imputation model usually results in estimates in the analysis model being biased. The variables must also be included in the imputation model in the right way: for example, Schafer noted that if interactions are omitted from the imputation model, then the estimated interactions in the analysis model would be biased toward the null.^7^

It is especially important to include in the imputation model the outcome variable for the analysis model.^5^^,^^11^ Failure to do so usually results in estimated regression coefficients for the analysis model also being biased toward the null. When the outcome in the analysis model is a survival or time-to-event outcome (eg, the outcome model is a Cox proportional hazards model) then there are 2 components to the outcome: a time-to-event variable denoting the time to the occurrence of the event or the time to censoring, and a binary indicator variable denoting whether the subject experienced the event or was censored. The recommended approach is to include both in the imputation model, with the time-to-event variable transformed using the cumulative survivor function.^16^ In addition, the imputation model is improved by including variables that are related to the missingness and variables that are correlated with variables of interest. In longitudinal data, when imputing a variable for a specific measurement occasion (eg, on the second clinic visit), one also needs to include in the imputation model future values of that variable (eg, the value of that variable at the third clinic visit).

### Imputing derived variables

The analysis model may include variables that are derived from other variables. Examples include body mass index (BMI, which is derived from height and weight), quadratic terms for continuous variables (eg, age^2^), and interactions between variables (ie, products of variables). When the component variables required to create the derived variable are missing (and therefore the derived variable is also missing), there are 2 main options for imputing the derived variables. The first option imputes the missing component variables and creates the derived variable after all variables have been imputed. Thus, for example, if height were missing, height would first be imputed and then combined with weight to create BMI. Von Hippel refers to this approach as “impute, then transform.” This approach is appealing, as it leads to derived variables that are consistent with the derivation rule. The obvious problem with the approach is that the derived variable is not part of the imputation model, so it may lead to bias, as explained in the preceding section (Which Variables to Include in the Imputation Model?). The second option is to treat the derived variable as simply another variable and to impute this variable directly. Thus, if height were missing (and thus BMI were also missing), height and BMI would be imputed for those subjects for whom they were missing. This approach is known as “transform, then impute”^17^ or “just another variable.”^11^ Note that the “just another variable” approach incorporates the components as well as the derived variable in the imputation model. This approach is appealing as it incorporates all necessary variables into the imputation model. However, it can lead to quadratic variables with negative values or BMI values that are inconsistent with the height and weight of the subject. It has been shown that in some settings the approach leads to accurate estimates of regression coefficients in the analysis model, though it can fail in others.^18^^,^^19^ Van Buuren describes some alternate strategies for specific types of dependencies.^10^ Because no strategy performs uniformly better, we may need some tailoring to the type of derived variable.

### Missing outcome variables

Multiple imputation is blind to which variables are outcomes and which variables are predictors in the final analysis model. When developing the imputation models, the important issue is to include in the imputation models all of the variables from the analysis model. This suggests that one can impute values of the outcome variable (for the analysis model) for those subjects for whom it is missing. However, von Hippel provided evidence that excluding subjects who are missing the outcome variable (for the analysis model) when fitting the outcome model will tend to be a better strategy.^20^ He proposed a strategy that he referred to as “multiple imputation, then deletion” (MID). Under MID, all subjects are used in the imputation process. Values are imputed for all missing data, including for those subjects who are missing the outcome variable. However, subjects for whom the outcome variable was imputed are then excluded when the analysis model is fitted in each imputed data set. The MID approach will tend to result in estimated regression coefficients for the analysis model that are more efficient (have smaller variability) than those obtained when fitting the analysis model in all subjects. In addition, the method is robust against bad imputation in the outcome. The MID procedure should not be used if there are auxiliary variables that are strongly related to the outcome (and not included in the analysis model) or if the scientific interest extends to parameters other than regression coefficients.^11^

## Case Study

We use data on patients hospitalised with heart failure in the province of Ontario to provide a case study illustrating the application of MI. The analysis model of interest is a logistic regression model in which death within 1 year of hospital admission is regressed on 10 patient characteristics.

### Data sources

We used data from the EFFECT (Enhanced Feedback for Effective Cardiac Treatment) study, which was an initiative to improve the quality of care for patients with cardiovascular disease in Ontario.^21^ We used data on 8,338 patients hospitalised with congestive heart failure from April 1, 2004, to March 31, 2005, at 81 Ontario hospital corporations. Data on patient demographics, vital signs and physical examination at presentation, medical history, and results of laboratory tests were collected on these patients by retrospective chart review. Subjects were linked to administrative health care data to determine vital status.

For the purposes of this case study, we considered 10 baseline covariates: age, respiratory rate at admission, glucose level, urea level, low-density lipoprotein (LDL) cholesterol level, sex, S3 (third heart sound) on admission, S4 (fourth heart sound) on admission, neck vein distension on admission, and cardiomegaly on chest X-ray. The first 5 are continuous and the last 5 binary. The outcome was a binary outcome denoting whether the patient died within 365 days of hospital admission. Logistic regression models for 30-day and 1-year mortality are often used in cardiovascular research.^22^, ^23^, ^24^ Our purpose here in using these data is to illustrate the application of statistical methods and not to draw clinical conclusions. Accurate estimation of the association of variables with cardiovascular outcomes in current patients may require the use of more recent data and a more comprehensive set of predictor variables. Furthermore, depending on the objective of the intended study, a different regression model may be more appropriate.

### Descriptive statistics

Means and percentages are reported for the continuous and binary variables, respectively, in Table 2. We also report the percentage of subjects with missing data for each of the variables. The percentage of missing data ranged from a low of 0% (age and sex) to a high of 73% (LDL cholesterol). Overall, 78% of subjects had missing data on at least 1 variable.

**Table 2:** Descriptive statistics of case study data

Variable	Mean (SD) or %	No. of subjects with observed data	No. of subjects with missing data	Percentage of subjects with missing data
Continuous variables				
Age, y	76.7 (11.6)	8338	0	0%
Respiratory rate at admission, breaths per minute	24.5 (7.0)	8138	200	2.4%
Glucose (initial lab test), mmol/L	8.6 (4.1)	8051	287	3.4%
Urea (initial lab test), mmol/L	10.3 (6.6)	8028	310	3.7%
LDL cholesterol, mmol/L	2.2 (0.9)	2272	6066	72.8%
Binary variables				
Female	50.9%	8338	0	0%
S3	6.2%	8126	212	2.5%
S4	2.7%	8135	203	2.4%
Neck vein distension	66.1%	7586	752	9.0%
Cardiomegaly on chest X-ray	47.7%	7711	627	7.5%
Outcome				
Death within 1 year	31.7%	8338	0	0%

LDL, low-density lipoprotein; S3, third heart sound; S4, fourth heart sound.

### Comparison of subjects with and without missing data

We conducted univariate comparisons of those with and without missing data. There are at least 2 reasons for these comparisons. First, as noted above, the imputation model is improved by including variables that are related to the missingness. These comparisons help to identify variables that should be included in the imputation model. Second, these analyses provide evidence as to the plausibility of the MAR assumption. If those with and without missing data differ on many observed variables, then it is plausible that they may also differ on unobserved variables. Note that a lack of significant univariate associations does not provide proof that the data are MCAR or MAR.

There were meaningful differences in age, sex, and mortality (the 3 variables that were not subject to missingness) between those with complete data and those with missing data. The average age of those with complete data was 73.7 years, and it was 77.5 years for those with missing data. Of those with complete data, 43.4% were female, while of those with missing data, 53.0% were female. Of those with complete data, 23.7% died within 1 year of admission, while of those with missing data, 33.9% died within 1 year of admission. Patients with missing data tended to be older, were more likely to be female, and more likely to die than those with complete data.

### Complete case analysis

**Figure 1:**
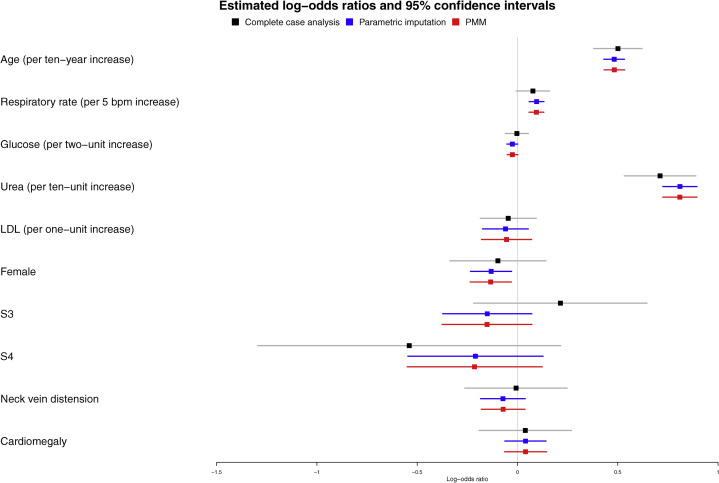
Estimated log-odds ratios and 95% confidence intervals for variables in the logistic regression model fit in the case study. There are 3 estimates/confidence intervals for each of the 10 variables: analyses using complete cases (**grey**); multiple imputation analyses when using parametric imputation (**blue**); and multiple imputation analyses when using predictive-mean matching (PMM) (**red**). LDL, low-density lipoprotein; S3, third heart sound; S4, fourth heart sound.

We conducted a complete case analysis restricted to the 1,806 subjects with complete data. The reason for doing this is that complete case analysis is less prone to user error than MI (because it does not rely on an imputation model) and we should be able to explain any differences between the complete case analysis and the MI analysis.^5^ We used logistic regression to regress death within 1 year of hospital admission on the 10 baseline covariates. The logarithm of the estimated odds ratios and associated 95% confidence intervals are reported in Figure 1 (log odds ratios are reported so that the confidence intervals are symmetric). Increasing age and urea were associated with an increased odds of death within 1 year and had 95% confidence intervals that excluded the null value. None of the binary variables had odds ratios whose associated 95% confidence interval excluded the null value. Note that the odds ratios for the 5 continuous variables are not directly comparable with one another, because they are measured on different scales.

### Multiple imputation

Imputation was conducted using the MICE algorithm with the use of PROC MI in SAS (SAS/STAT v14.1). Logistic regression models were used as the imputation models for the binary variables, and linear regression models were used as the imputation models for the continuous variables. All variables (including the binary outcome variable) were included in each imputation model (with the obvious exception of the variable that was being imputed). Using the rule of thumb suggested by White et al., we created 78 imputed data sets because 78% of the subjects had any missing data. For comparative purposes, we used von Hippell’s 2-stage algorithm with 10 imputed data sets in the first stage with the criterion that the standard errors of the estimated regression coefficients be estimated accurately to 2 decimal places. The algorithm suggested that 80 imputed data sets were necessary to estimate the standard error of the intercept term with the desired precision and that at most 15 imputed data sets were necessary to estimate the standard errors of the 10 covariates with the desired precision.

As a sensitivity analysis we used PMM when imputing missing values for the continuous variables. Software code for conducting these analyses is provided in Supplemental Appendix S1 (SAS code), Supplemental Appendix S2 (R code), and Supplemental Appendix S3 (Stata code).

### Descriptive statistics in the imputed data sets

**Figure 2:**
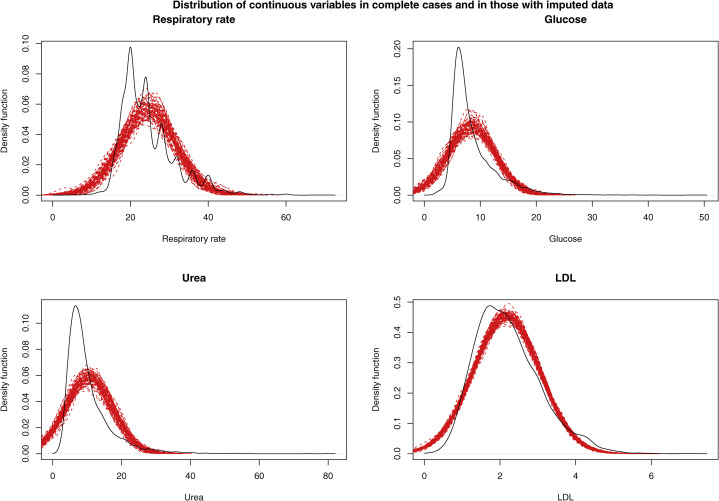
Distribution of continuous variables in complete cases and in those with imputed data when using parametric imputation. The **solid black line** represents the distribution of the given continuous variable in those subjects for whom that variable was not missing. The **dashed red lines** denote the distribution of the imputed value for that variable in those subjects for whom the variable was missing. There is 1 red line for each of the imputed data sets. LDL, low-density lipoprotein.

**Figure 3:**
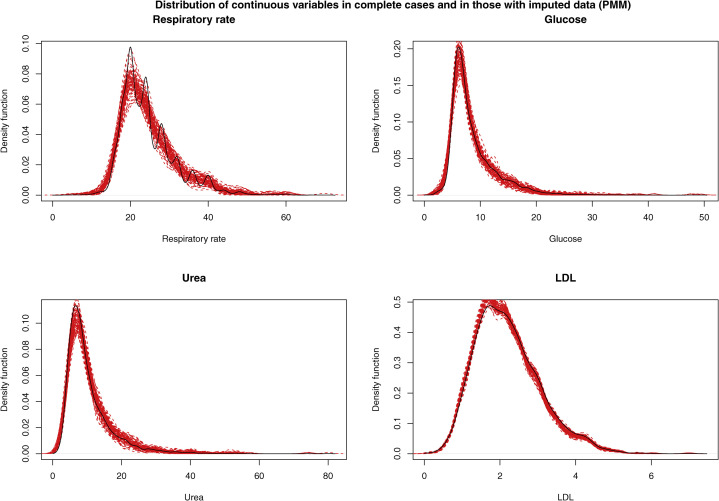
Distribution of continuous variables in complete cases and in those with imputed data when using predictive mean matching (PMM). The **solid black line** denotes the distribution of the given continuous variable in those subjects for whom that variable was not missing. The **dashed red lines** denote the distribution of the imputed value for that variable in those subjects for whom the variable was missing. There is 1 red line for each of the imputed data sets. LDL, low-density lipoprotein.

Nonparametric density plots were used to describe the distribution of the 4 continuous variables that were subject to missing data in the complete cases and in those subjects who were missing data for the given continuous variable. The latter was done separately in each of the imputed data sets. These are described in Figure 2 (parametric imputation) and Figure 3 (PMM). The density function in the complete cases is shown as a solid black line, and the density function of the imputed variable in each of the imputed data sets is shown with a dashed red line. When using parametric imputation, the distribution of imputed respiratory rate, glucose, and urea failed to display the skewness seen in subjects for whom the variable was observed. However, the distribution of imputed values of LDL was similar to the empirical distribution in subjects for whom LDL was measured. When using PMM, the distribution of the imputed values tended to be very similar to that of the observed values of the variable.

### Logistic regression in the imputed data sets

In each imputed data set, we regressed the binary outcome denoting death within 1 year of hospital admission on the 10 covariates described in Table 2. The regression coefficients and their standard errors were pooled using Rubin’s rules. The estimate of the Monte Carlo error for the 10 estimated regression coefficients ranged from 0.000042 for age to 0.005502 for LDL cholesterol. Thus, if we repeated the entire imputation process multiple times, we would expect to see only minor variation in the estimated regression coefficients.

The log odds ratios and their associated 95% confidence intervals obtained using parametric imputation are shown in Figure 1. Three continuous variables (age, respiratory rate, and urea) had a positive association with 1-year mortality, while females had a lower risk of death than males. The odds ratios and associated 95% confidence intervals obtained using PMM imputation are also shown in Figure 1. The estimated odds ratios and associated confidence intervals obtained using PMM imputation were essentially identical to those obtained using parametric imputation. In comparing the results of the 3 regression analyses, one observes that the confidence intervals obtained from the imputation-based analyses were narrower than those obtained in the complete case analysis. For some variables (eg, age, S3, and S4), the confidence intervals obtained using the complete case analysis were substantially wider than those obtained using MI.

## Discussion

Missing data occurs frequently in clinical research. MI is a statistical tool that allows the researcher to replace missing values with multiple plausible values of the variable in question. The use of MI allows the researcher to analyse complete data sets while incorporating the uncertainty in the imputed values of the variable. We have provided a brief introduction to MI and guidance regarding its implementation. We illustrated the application of MI through the analysis of data on patients hospitalised with heart failure.

When applying MI, researchers should explore differences between the observed and imputed distributions and between the complete case analyses and the MI analyses. We refer readers to previously published guidelines for reporting analyses affected by missing data.^5^^,^^25^

This introduction to MI was not intended to be exhaustive. We refer the interested reader to several excellent texts on MI^1^, ^2^, ^3^^,^^10^ as well as to more detailed overview articles.^7^^,^^11^ We have focused our attention on MI in observational studies in which clustering of subjects or a multilevel structure is absent. Other works describe methods for using MI with multilevel data.^10^^,^^26^, ^27^, ^28^, ^29^ Similarly, we have focused on the use of parametric models (eg, logistic regression models or linear regression models) for the imputation models. An area of current research is on the use of machine-learning methods for MI.^30^ We have focused on the use of MI when data are either MCAR or MAR. The described methods must be modified if it is thought that the data are MNAR. Van Buuren summarises different methods to address data that are MNAR.^10^ The simplest approach is to assume that the distribution of a variable in those with missing data is shifted compared with the distribution in those with complete data. Sensitivity analyses can be conducted in which the magnitude of the shift parameter is allowed to vary.

We have focused on the MICE algorithm for MI, along with a modification, PMM. This is not the only method to impute missing data. An earlier method has been described as “joint modeling,”^10^ of which MI under a normal model is a specific implementation.^4^ This approach assumes that the set of variables follow a joint multivariate distribution. The multivariate normal distribution is widely used in applications.^10^ Under this implementation, the variables are assumed to follow a multivariate normal distribution. Once the parameters of this distribution have been estimated, missing values can be imputed by random draws from this multivariate distribution. In theory, this approach requires that all of the variables be continuous. In practice, binary or categorical variables frequently occur (eg, presence or absence of diabetes). Schafer and Graham suggest that despite this theoretical limitation, they have found the multivariate normal distribution to be useful in a wide range of settings.^4^ Furthermore, they provide suggestions for incorporating binary and categorical variables as well as nonnormally distributed continuous variables. However, others have suggested that these methods of incorporating noncontinuous variables may not perform as desired.^10^ Given the flexibility of the MICE algorithm and its ability to explicitly incorporate different types of variables, its use may be attractive to researchers in biomedical research.

In our case study, we obtained similar parameter estimates when using parametric imputation as when using PMM imputation. This is to be expected for estimates that depend on the middle of the distribution, such as means or regression coefficients. In practice, it may be difficult to provide examples where PMM imputation beats a well crafted parametric imputation model. However, in practice, analysts often prefer PMM imputation because it preserves typical features in the raw data. For example, it accounts for discreteness of data, avoids impossible values, preserves location of quantiles, and is highly robust to imputation model misspecification. All this costs no additional work on the part of the analyst. If the complete-data model depends on such features, then the inference will also be better when using PMM imputation.

In this tutorial article we have focused on the use of MI in observational studies. In randomised controlled trials (RCTs), MI is not always the optimal approach.^6^ When a univariate outcome is MAR, a complete case analysis using an adjusted analysis is unbiased and efficient.^6^ With a multivariate outcome (eg, an outcome measured at multiple occasions over the course of follow-up), the use of a linear mixed model with missing data in the outcome only will tend to result in estimates with smaller standard errors compared with the use of MI.^6^ If MI is used, it is suggested that imputation be conducted separately in the different arms of the trial.^6^

In summary, MI replaces missing values with plausible values. By creating multiple imputed data sets, the analyst can explicitly account for the uncertainty inherent in the imputed values. Historical approaches such as complete case analysis, mean imputation, and single imputation potentially result in bias, incorrect estimates of standard errors, and consequently incorrect tests of statistical significance. Researchers are encouraged to consider MI as an important tool to address the problems associated with missing data in clinical research.

## Funding Sources

This study was supported by the ICES which is funded by an annual grant from the Ontario Ministry of Health and Long-Term Care (MOHLTC). The opinions, results and conclusions reported in this paper are those of the authors and are independent from the funding sources. No endorsement by ICES or the Ontario MOHLTC is intended or should be inferred. The data sets used for this study were held securely in a linked deidentified form and analysed at ICES. Although data-sharing agreements prohibit ICES from making the data set publicly available, access may be granted to those who meet prespecified criteria for confidential access, as described at https://www.ices.on.ca/DAS. This research was supported by a operating grant from the Canadian Institutes of Health Research (CIHR) (grant number MOP 86508). The EFFECT data used in the study was funded by a CIHR Team Grant in Cardiovascular Outcomes Research (grant numbers CTP79847 and CRT43823). P.C.A. and D.S.L. are supported in part by Mid-Career Investigator awards from the Heart and Stroke Foundation. D.S.L. is supported by the Ted Rogers Chair in Heart Function Outcomes. I.R.W. was supported by the Medical Research Council Programme MC_UU_12023/21.

## Disclosures

The authors have no conflicts of interest to disclose.
