# Supplementary material for: Missing Data in Clinical Research: A Tutorial on Multiple Imputation
Source: Can J Cardiol. 2021 Sep;37(9):1322–31. doi: 10.1016/j.cjca.2020.11.010 (PMC8499698; doi:10.1016/j.cjca.2020.11.010)
Supplement: Supplementary Material [file mmc1.docx]

**Supplemental Appendix S1. SAS code for multiple imputation**

* **This code is provided for illustrative purposes and comes**

**with absolutely no warranty**;

* The dataset used for these analyses cannot be publicly

Distributed. Please do not contact the authors requesting

the dataset;

* MI using the default parametric imputation for the continuous variables;

* In the dataset are the following variables:

age: patient age (years)

resp: Respiratory rate

glucose: Glucose

urea: Urea

ldl: LDL cholesterol level

female: Binary variable (female=1/male=0)

s3: S3 (third heart sound)

s4: S4 (fourth heart sound)

neckvdis: Neck vein distension

cmg: Cardiomegaly on chest X-ray

mort1yr: Binary variable denoting death within one year;

proc mi data=cohort seed=2122019 nimpute=pctmissing (max=99)

out=tutorial_mi;

class female s3 s4 neckvdis cmg mort1yr;

fcs plots=trace nbiter=20 logistic(mort1yr female s3 s4 neckvdis cmg);

var mort1yr age female resp s4 s3 glucose urea cmg neckvdis ldl;

run;

* MI using PMM for the continuous variables;

proc mi data=cohort seed=2122019 nimpute=pctmissing (max=99)

out=tutorial_mi_pmm;

class female s3 s4 neckvdis cmg mort1yr;

fcs plots=trace nbiter=20 logistic(mort1yr female s3 s4 neckvdis cmg);

fcs regpmm(age resp glucose urea ldl);

var mort1yr age female resp s4 s3 glucose urea cmg neckvdis ldl;

run;

* Analyses in the imputed datasets;

Proc sort data=tutorial_mi; by _imputation_; run;

Proc logistic data=tutorial_mi descending;

Model mort1yr = age resp glucose urea ldl female s3 s4 neckvdis cmg

/covb;

By _imputation_;

Ods output ParameterEstimates=lgsparms covB=lgscovb;

Run;

* Pooling results using Rubin’s Rules;

Proc mianalyze parms=lgsparms covb(effectvar=stacking)=lgscovb;

Modeleffects Intercept age resp glucose urea ldl female s3 s4

neckvdis cmg;

ods output ParameterEstimates=MI;

run;

proc print data=MI;

run;

**Supplemental Appendix S2. R code for multiple imputation**

**# This code is provided for illustrative purposes and comes**

**with absolutely no warranty;**

################################################################################

# Read in data.

################################################################################

zlist<- list(age=0,resp=0,glucose=0,urea=0,ldl=0,female=0,s3=0,s4=0,neckvdis=0,

cmg=0,mort1yr=0)

cohort <- data.frame(scan("mi_tutorial.txt",zlist))

data <- cohort[,c("mort1yr","age","female","resp","s4","s3",

"glucose","urea","cmg","neckvdis","ldl")]

################################################################################

# MI using the parametric imputation for the continuous variables

################################################################################

meth <- make.method(data)

meth[meth == "pmm"] <- "norm"

nimp <- 100 * nic(data) / nrow(data)

imp.parm <- mice(data,m=nimp,method=meth,maxit=20,seed=2122019)

plot(imp.parm)

# Analyses in the imputed datasets

fit.parm <- with(imp.parm,glm(mort1yr ~ age + resp + glucose +

urea + ldl + female + s3 + s4 + neckvdis + cmg,family = "binomial"))

# Pooling results using Rubin's Rules

summary(pool(fit.parm), confint = TRUE, exponentiate = TRUE)

################################################################################

# MI using the default PMM for the continuous variables

################################################################################

imp.pmm <- mice(data,m=nimp,maxit=20,seed=2122019)

plot(imp.pmm)

# Analyses in the imputed datasets

fit.pmm <- with(imp.pmm,glm(mort1yr ~ age + resp + glucose +

urea + ldl + female + s3 + s4 + neckvdis + cmg,family = "binomial"))

# Pooling results using Rubin's Rules

summary(pool(fit.pmm), confint = TRUE, exponentiate = TRUE)

**Supplemental Appendix S3. Stata code for multiple imputation**

*** This code is provided for illustrative purposes and comes**

**with absolutely no warranty;**

infile age resp glucose urea ldl female s3 s4 neckvdis cmg mort1yr using "mi_tutorial.txt"

set seed 2122019

mi set flong

mi register imputed resp glucose urea ldl // continuous and incomplete

mi register imputed s3 s4 neckvdis cmg // binary and incomplete

mi register regular mort1yr age female // complete

* MI using parametric imputation for the continuous variables;

*mi impute chained (logit) s3 s4 neckvdis cmg (regress) resp glucose urea ldl = mort1yr age female, add(20)

* alternative MI using PMM for the continuous variables;

mi impute chained (logit) s3 s4 neckvdis cmg (pmm, knn(10)) resp glucose urea ldl = mort1yr age female, add(20)

mi estimate: logistic mort1yr age resp glucose urea ldl female s3 s4 neckvdis cmg
